# Creatine and Nicotinamide Prevent Oxidant-Induced Senescence in Human Fibroblasts

**DOI:** 10.3390/nu13114102

**Published:** 2021-11-16

**Authors:** Avinash S. Mahajan, Venkata S. Arikatla, Anita Thyagarajan, Tetyana Zhelay, Ravi P. Sahu, Michael G. Kemp, Dan F Spandau, Jeffrey B. Travers

**Affiliations:** 1 Department of Pharmacology & Toxicology, Boonshoft School of Medicine at Wright State University, Dayton Ohio, OH 45435, USA; mahajan.28@wright.edu (A.S.M.); arikatla.3@wright.edu (V.S.A.); anita.thyagarajan@wright.edu (A.T.); zhelay@gmail.com (T.Z.); ravi.sahu@wright.edu (R.P.S.); mike.kemp@wright.edu (M.G.K.); 2 Dayton Veterans Administration Medical Center, Dayton Ohio, OH 45428, USA; 3 Departments of Dermatology and Biochemistry & Molecular Biology, Indiana University School of Medicine, Indianapolis, IN 46223, USA; dspandau@iu.edu; 4 Richard L. Roudebush Veterans Administration Medical Center, Indianapolis, IN 46202, USA

**Keywords:** fibroblast, senescence, reactive oxygen species, insulin-like growth factor-1, Cr, NAM

## Abstract

Dermal fibroblasts provide structural support by producing collagen and other structural/support proteins beneath the epidermis. Fibroblasts also produce insulin-like growth factor-1 (IGF-1), which binds to the IGF-1 receptors (IGF-1Rs) on keratinocytes to activate signaling pathways that regulate cell proliferation and cellular responses to genotoxic stressors like ultraviolet B radiation. Our group has determined that the lack of IGF-1 expression due to fibroblast senescence in the dermis of geriatric individuals is correlated with an increased incidence of skin cancer. The present studies tested the hypothesis that pro-energetics creatine monohydrate (Cr) and nicotinamide (NAM) can protect normal dermal human fibroblasts (DHF) against experimentally induced senescence. To that end, we used an experimental model of senescence in which primary DHF are treated with hydrogen peroxide (H_2_O_2_) in vitro, with senescence measured by staining for beta-galactosidase activity, p21 protein expression, and senescence associated secretory phenotype cytokine mRNA levels. We also determined the effect of H_2_O_2_ on IGF-1 mRNA and protein expression. Our studies indicate that pretreatment with Cr or NAM protects DHF from the H_2_O_2_-induced cell senescence. Treatment with pro-energetics post-H_2_O_2_ had no effect. Moreover, these agents also inhibited reactive oxygen species generation from H_2_O_2_ treatment. These studies suggest a potential strategy for protecting fibroblasts in geriatric skin from undergoing stress-induced senescence, which may maintain IGF-1 levels and therefore limit carcinogenesis in epidermal keratinocytes.

## 1. Introduction

Senescence is defined as the state of a cell when a series of degenerative events occur, followed by maturation, and is associated with the process of aging [1]. Various dysfunctional processes like DNA damage, oxidative stress, telomere shortening, and other organizational modifications of the chromatin are major factors causing senescence in a cell [2,3,4]. A distinctive feature of a senescent cell is the irreversible arrest of cell cycle predominantly in the G1 phase and a lack of response towards growth factors [5]. Apoptosis is resisted in senescent cells and both quantitative and qualitative alterations of expression levels of various genes are observed [6]. The cyclin-dependent kinase inhibitor p21 is highly expressed in senescent cells, in a p53-dependent manner, and limits cell cycle progression, hence affecting cell proliferation [7]. Senescent cells also contain high levels of the enzyme beta-galactosidase (β-gal) [8]. Certain inflammatory cytokines including IL-6, IL-8, and TNF-α, termed the senescence-associated secretory phenotype (SASP), are expressed in large amounts in the senescent cell [9]. When these cytokines are released in the microtissue environment, they attract immune cells to specific locations to potentially eliminate senescent cells [10]. However, this phenomenon is not commonly seen in geriatric tissues which potentially leads to increased numbers of senescent cells in the geriatric microenvironment. Multiple factors, as mentioned, determine the fate of a cell and in certain conditions accelerate senescent cell formation. 

Dermal fibroblasts synthesize connective tissue and insulin-like growth factor-1 (IGF-1) [11,12]. Tyrosine kinase IGF-1 receptors (IGF-1R) expressed by skin keratinocytes are activated by IGF-1 secreted by dermal fibroblasts. As a result, several pathways responsible for cell proliferation, proliferation, and apoptosis are initiated. However, in geriatric skin, many dermal fibroblasts have undergone senescence. This age-associated phenomenon impairs the ability of the cells to secrete IGF-1 and causes human keratinocytes to respond to UVB inappropriately [13]. DNA damage checkpoint signaling and nucleotide excision repair are found to be compromised in human keratinocytes with inactive IGF-1 receptors [14]. The keratinocytes accumulate DNA damage when exposed to stressors like UVB or other pro-oxidative agents. Persistent (unrepaired) DNA damage can cause malignancy [15].

Creatine (Cr) is a nitrogenous organic acid and primarily found in food via meat sources. While mostly found in muscles, trace amounts of creatine have been found in brain and testes. Cr can be endogenously synthesized from amino acids in the kidney and liver, producing about 1–3 g/day in the body [16]. It is a proenergetic, as its major function is to form phosphor-creatine which when acted upon by creatine kinase can transfer the high energy phosphate to ADP creating ATP which provides energy [16,17]. Previous studies have shown that Cr elevates IGF-1 mRNA expression levels in skeletal muscle in adult humans [18,19]. The anticatabolic effects of Cr on various cell types can serve as an antioxidant and reduce ROS levels [20]. Its benefits have been well known in muscle cells and is being applied in the field of sports and nutrition. Cr has also shown to play protective effects against mitochondria degradation, and age-related neurological disorders [20]. 

Nicotinamide (NAM) is a vitamin B3 amide derivative. It cannot be endogenously synthesized, hence is obtained from exogenous sources, mostly from the diet [21,22]. NAM is the precursor of NAD+ which is a coenzyme that participates in the salvage pathway [22]. It is known to increase the synthesis of NAD+. NAM has been shown to exert positive effects on cell survival in many cell types, facilitate fetal cell maturation, and cause differentiation of embryonic stem cells to cells producing insulin [23]. In the case of ischemic infarction, NAM pretreatment has been reported to protect brain cells, likely by inhibiting oxidative stress [24]. NAM treatment of human primary fibroblasts resulted in increased cellular lifespan and exerts protective effects against ROS levels [25,26]. However, the effects of these agents in protecting against senescence are presently not well understood. 

## 2. Materials and Methods

### 2.1. Reagents

All routine reagents including DCFH2-DA were obtained from Sigma-Aldrich Co. (St. Louis, MO, USA). 

### 2.2. Cell Culture

Primary cultures of dermal human neonatal fibroblasts (DHF) were obtained from neonatal skin discarded from foreskin tissue. This collection of foreskin tissue is deemed “Research not subject to FDA or common rule definitions of human subjects research”. Approval by the Indiana University School of Medicine IRB #NS0812-03. DHF were grown in low glucose Dulbecco’s modified Eagle’s medium (Hyclone) supplemented with 10% fetal bovine serum and 5% penicillin/streptomycin. Tert-immortalized fibroblasts (Tert-HF; NHF1 cells) were obtained from William Kaufmann at the University of North Carolina and were grown in high glucose Dulbecco’s modified Eagle’s medium (Hyclone) supplemented with 10% fetal bovine serum and 5% penicillin/streptomycin. Cells were kept in 5% CO_2_ and 20% O_2_ conditions. 

### 2.3. Treatments

DHF cells were grown in 10 cm dishes until approximately 80% confluent. Media was removed and cells washed three times with PBS. For some experiments, cells were plated on 10 cm or 6 cm or 3.5 cm plates and were treated with various concentrations of H_2_O_2_ and Cr or NAM or both (Sigma Aldrich). In other experiments, cells were treated with Cr or NAM or both 24 h post plating and kept in the incubator overnight. Cells were treated with 600 µM H_2_O_2_ for 2 h. The cells were then washed thrice with PBS and re-treated with Cr or NAM or both. In addition, 72 h post H_2_O_2_ treatment, media was removed, cells were washed twice with PBS 1X, and harvested for performing assays. H_2_O_2_ was diluted at desired concentrations directly in growth media, whereas Cr and NAM were dissolved in autoclaved water followed by sterile filtration using a 0.2 µm filter to obtain various concentrations. 

### 2.4. Cell Proliferation (MTT Assay)

Cells were treated with varying concentrations of agents upon reaching approximately 50% confluency. H_2_O_2_ exposure was for 2 h whereas Cr and NAM were exposed to the cells until the time of harvest. Media was removed post 24/48/72 h of treatment. In addition, 1.25 mL of 5 mg/mL MTT solution was added to 23.75 mL of Epilife medium (for 0.25 mg/mL MTT final concentration MTT final concentration). Two ml of this MTT-containing media was added to each plate and incubated for 45 min at 37 °C. Media was removed and replaced with 1 mL of DMSO to solubilize MTT dye and was well mixed. In addition, 100 μL of the resulting solution was transferred to a 96-well plate, three wells per sample. Using a Synergy H1 plate reader (Bio-Tek), absorbance was read at 570 nm. Cell proliferation was calculated by an average of three identical wells and normalized to the no treatment group.

### 2.5. Experimental Model of Senescence

Cells underwent experimentally induced senescence with H_2_O_2_. HDF at 200,000 cells/100 mm plate, 100,000 cells/60 mm plate, and 30,000 cells/35 mm plate were plated using fresh media. A volume of 10 mL, 5 mL, and 2 mL were constantly maintained in 100 mm, 60 mm, and 35 mm plates, respectively. These cells were incubated for 24 h in optimum conditions. Then, cells were pretreated with water vehicle or Cr or NAM in fresh media and incubated for 24 h. The cells were treated with H_2_O_2_ in fresh media. After 2 h, cells were washed twice with 1X PBS and fresh media containing vehicle or Cr or NAM treatment was added. Cells were incubated for 72 h post treatments and were utilized for further assays. In some experiments, cells were not pretreated with Cr or NAM or both but given 2 h after H_2_O_2_ treatment. 

### 2.6. Beta Galactosidase Staining 

Cells were grown in 35 mm culture plates. A senescence detection kit (ab65351, abcam) was used to stain the cells for β-gal, and images were captured using a BioTek Cytation 5 Cell Imaging Multi-Mode Reader.

### 2.7. Western Blot

To observe protein expression, cells that were grown on 10 cm culture plates were harvested by scrapping and then lysed in RIPA buffer. The amount of protein was quantified using Bradford reagent. Ten μg of protein were obtained from each treatment group and added to a 1.5 mL centrifuge tube. To this 4 μL of 6X, SDS was added. The volume was brought up to 25 μL with RIPA buffer. The mixture was boiled at 95 °C for 5 min and centrifuged at max speed for 30 s. A 25 μL aliquot of this mixture was added to each well and gel electrophoresis was conducted for 45 min at 200 V. The gel was removed and soaked in semi-dry transfer buffer. Alongside, nitrocellulose membrane and thick blot paper were soaked in transfer buffer. A sandwich was prepared in the transfer unit in the order of blot paper, membrane, gel, and blot paper. The transfer was set at 25 V/2.5 Amps for 15 min. Next, the membrane was briefly washed thrice with 1X TBST. In addition, 0.5% ponceau stain was added and rocked on the shaker for 2 min to observe the protein bands on the membrane. Brief washes were then performed three times. Ten milliliters of 5% milk in TBST were added and rocked on the shaker for 30 min to allow blocking. Four brief washes with 1X TBST were followed. Varying dilutions of primary antibody were used based on the protein of interest; Actin: 1:20,000 (Rb anti-cytoskeletal actin, Bethyl), p21: 1:1000 (Mouse IgG 2a, ab91361, abcam), IGF-1: 1:2500 (Rb pAB to IGF-1, ab9572, abcam). The membrane was rocked with primary antibody overnight at 4 °C. Primary antibody was removed and four 5-min washes with 1X TBST were given. Then, the membrane was rocked for 1 h with secondary antibody 1:5000; Actin- Polyclonal goat Anti-rabbit immunoglobin/HRP, Dakocytomation, p21-Goat Anti-mouse IgG, Invitrogen, IGF-1-Goat pAB to Rb IgG, Abcam). Five, brief, 5-min washes were followed, and the membrane was exposed to ECL substrate (Clarity Max, Bio-Rad, CA, USA) and visualized using a Molecular Imager Chemi-Doc XRS+ imaging system (Bio-Rad). Datasets were quantified relative to actin and normalized to a reference point (sham or H_2_O_2_). 

### 2.8. RT-qPCR

Media was removed, and the cells were washed with PBS 1X. Cells were trypsinised with 1 mL 0.05% trypsin-EDTA for 5 min at 37 °C in the incubator. Four milliliters of fresh media were added. The cells were transferred in a conical tube and centrifuged at 3000 rpm for 5–10 min. Cell pellets were collected after discarding the supernatant. Cells were disrupted by adding 350 μL of RLT buffer. An RNA isolation kit (RNeasy Plus Mini Kit, Qiagen, MD, USA) was used to elute RNA from cells. RNA concentration was determined using a Nanodrop spectrophotometer. Equal amounts of RNA were pipetted. An RT kit (QuantiTect, Reverse Transcription Kit, Qiagen) was used to convert RNA to cDNA. Twelve microliters of samples were pipetted in an 8-tube strip and 2 μL of 7x genomic DNA wipeout buffer were added. This was incubated in the thermocycler at 42 °C for 5 min. The samples were kept on ice, and a master mix of the reverse transcription was prepared. For one reaction, the master mix requires 4 μL of RT buffer, 1 μL of primer, and 1 μL of RT enzyme. Each reaction was added to each tube of the 8-tube strip. This was then incubated for 15 min at 42 °C followed by a 5-min incubation at 95 °C. To perform qPCR, for one reaction, 5 μL of 2X master mix (TaqMan™ Fast Universal PCR Master Mix (2X), no AmpErase™ UNG), 0.5 μL of 20X Taqman primer (IGF-1-Hs01547656_m1, IL6-Hs00174131_m1, IL8- Hs00174103_m1, TNF-α-Hs00174128_m1, B2M-Hs00187842_m1; Taqman gene expression assays, ThermoFisher Scientific, MA, USA), 0.5 μL of RT reaction, and 3 μL of molecular biology grade water (Ultrapure Water for Molecular Biology, MilliporeSigma, St. Louis, MO, USA) were added to each well of a 96-well plate. In this study, Beta-2-microglobulin was used as the reference. The 96-well plate was placed in the Bio-Rad Q-PCR machine for 40 cycles (1 cycle-95 °C for 3 min, 95 °C for 30 s, and 55 °C for 30 s). C_t_ values were obtained from the software and then the Livak method or the ΔΔCt method were used to calculate the expression fold change relative to the control.

### 2.9. Measurement of ROS

To measure intracellular ROS, the non-fluorescent probe 2′,7′-dichlorodihydrofluorescein diacetate was used (DCFH2-DA; Sigma-Aldrich Co., LLC, St. Louis, MO, USA). In addition, 200,000 cells were cultured in a 60 mm plate, pretreated for 24 h with DMEM containing various concentrations of Cr and NAM, washed twice with PBS, and incubated for 30 min in the dark at 37 °C with 5 µM of DCFH2-DA. The cells were then washed 3–4 times with PBS before being incubated with H_2_O_2_ for 30 min. Then, the cells were washed twice again with HBSS before being examined under a fluorescence microscope using the BioTek Cytation 5 machine, and the mean fluorescence intensity was measured by using ImageJ software. In some experiments, superoxide dismutase (SOD) enzyme activity levels were assayed using a kit and following manufacturer’s directions (SOD Assay kit-WST [Dojindo, Inc., Gaithersburg, MD, USA]).

### 2.10. SOD Activity 

Superoxide dismutase activity was assessed using an SOD activity test kit (SOD Assay kit-WST [Dojindo, Inc.]). Furthermore, 800,000 cells were cultured in 100 mm plate, pretreated for 24 h with DMEM containing various concentrations of Cr and NAM, washed twice with PBS, and incubated with H_2_O_2_ for 30 min. Then, the cells were washed twice again with PBS and scrap the cells after adding 5 mL of PBS. Collect the cells in 15 cm conical tube and centrifuge for 5 min at 450 rpm. After that, discard the PBS and add 800 mL of lysis buffer to the cell pellet. Superoxide dismutase (SOD) enzyme activity levels were assayed using a kit and following the manufacturer’s directions.

### 2.11. Statistical Analysis

Groups for MTT assay were compared and analyzed using two-way ANOVA, where significance was considered if *p* < 0.05. Beta-gal staining, Western blot, and RT qPCR were quantified and analyzed using one-way ANOVA, where significance was considered if *p* < 0.05.

## 3. Results

### 3.1. Effects of Agents on Cell Proliferation

**Figure 1:**
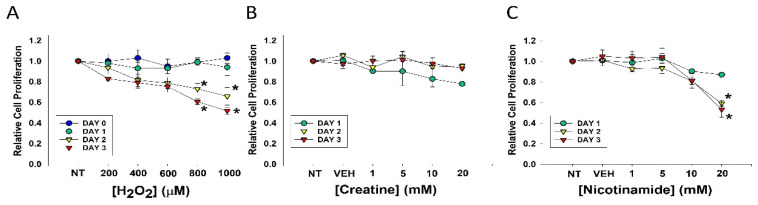
Effects of varying concentrations of agents on fibroblast cell proliferation. DHF were treated with various concentrations of (**A**) H_2_O_2_; (**B**) Cr; (**C**) NAM, water (1%) vehicle, or no treatment (sham; NT) and MTT assays performed at days 1, 2, or 3 to assess cellular viability. The data are mean ± SE of triplicate experiments. * Statistically significant (*p* < 0.05) decreased relative cell proliferation was found on days 2 and 3 of 800 μM and 1 mM H_2_O_2_ treatment, as well as 20 mM NAM at days 2 and 3.

Hydrogen peroxide is commonly used to experimentally induce oxidative stress in cells, which is a major senescence-generating stimulus [26,27]. To observe potential toxic effects, DHF cells were treated with varying doses of H_2_O_2_, Cr, and NAM. As shown in Figure 1A, fibroblasts were relatively resistant to the toxic effects of these agents.

Decreased cell proliferation was observed three days after treatment with levels of 800 or 1000 μM. In contrast, treatment with Cr up to 20 mM and NAM up to 10 mM had no discernable toxic effects on DHF under these conditions (Figure 1B,C). However, MTT assays revealed NAM at 20 mM resulted in significant cellular toxicity as denoted by growth inhibition (Figure 1C). These studies provided the premise for our use of 600 μM H_2_O_2_ and Cr and NAM concentrations at 10 mM and below. 

### 3.2. Effects of Cr and NAM on Oxidant Senescence

**Figure 2:**
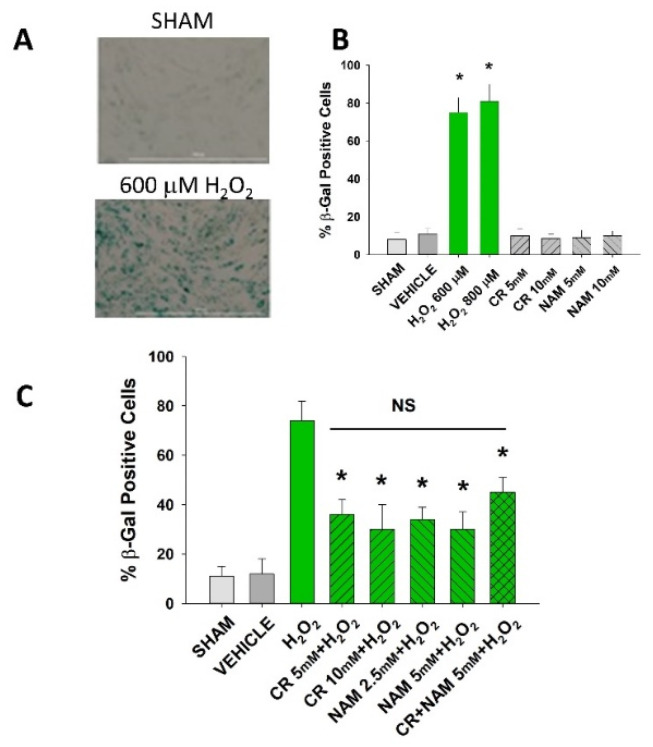
Effect of Cr and NAM pretreatment on oxidant-induced β-gal activity. DHF were exposed to various concentrations of Cr or NAM alone or combined (5 mM each), media alone (SHAM), or water vehicle (1%). Twenty-four hours later, the cells were exposed to 600 µM H_2_O_2_ for 2 h, followed by replacement with treatment and harvesting 72 h later for β-gal staining. (**A**) representative pictures of β-gal staining of vehicle versus H_2_O_2_ treatment, indicating increased staining (senescence) due to oxidant exposure; (**B**,**C**) % β-gal positive cells in response to various treatments. The data are mean ± SE from 3–4 separate experiments using duplicate samples. * Denotes statistically significant changes in the Cr/NAM treated levels in comparison to H_2_O_2_ alone. NS, the treatments compared to one another are not statistically significantly different.

**Figure 3:**
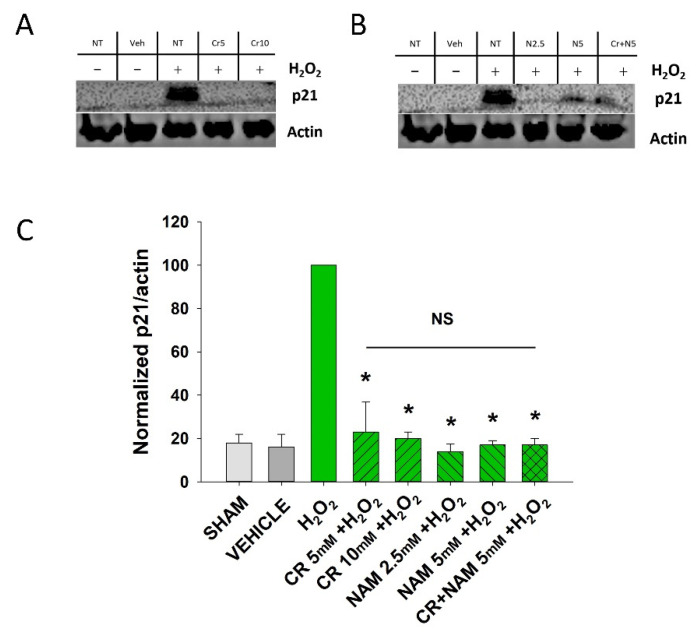
Effect of Cr and NAM on oxidant induced p21 levels. DHF were exposed to various concentrations of 10 mM Cr or 2.5 or 5 mM NAM alone or combined (5 mM each), media alone (SHAM), or water vehicle (1%). Twety-four hours later, the cells were exposed to 600 µM H_2_O_2_ for 2 h, followed by harvesting 72 h later for p21 protein analysis. (**A**,**B**) representative Western blotting indicating that H_2_O_2_ treatment increased p21 protein expression, whereas pretreatment with (**A**) Cr and (**B**) NAM did not result in increased p21 expression levels; (**C**) graphical summary of Western blot images normalized to actin. The data are mean ± SE from 3–4 separate experiments using duplicate samples. Data sets of p21 levels were quantified relative to actin and normalized to an H_2_O_2_ group. * Denotes statistically significant changes in the Cr/NAM treated levels in comparison to H_2_O_2_ alone. NS, the treatments compared to one another are not statistically significantly different.

Senescent cells are characterized by increased β-gal activity as well as increased p21 levels [27,28,29,30]. As shown in Figure 2, a 2 h treatment with 600 μM H_2_O_2_ resulted in increased β-gal activity 72 h post-treatment. However, Cr or NAM treatment alone did not result in increased numbers of β gal-positive cells (Figure 2B). Pre-treatment with Cr or NAM for two days prior to H_2_O_2_ exposure resulted in decreased numbers of β-gal-positive cells (Figure 2). Of note, combining 5 mM of Cr + NAM did not result in additional protection against oxidant senescence over the agents given individually (Figure 2C). Consistent with the β-gal data, treatment with H_2_O_2_ also resulted in increased levels of p21 protein (Figure 3). Moreover, pretreatment with Cr or NAM blocked the H_2_O_2_-induced elevations in p21 levels. Of interest, concentrations of Cr at 2.5 mM and NAM at 1 mM had no effect on oxidant-induced senescence (data not shown). These studies indicate that H_2_O_2_ treatment results in DHF expressing two separate markers of senescence, which is diminished by pre-treatment with Cr or NAM. 

### 3.3. Effects of Cr and NAM Pretreatment on Fibroblast Cytokine Levels 

**Figure 4:**
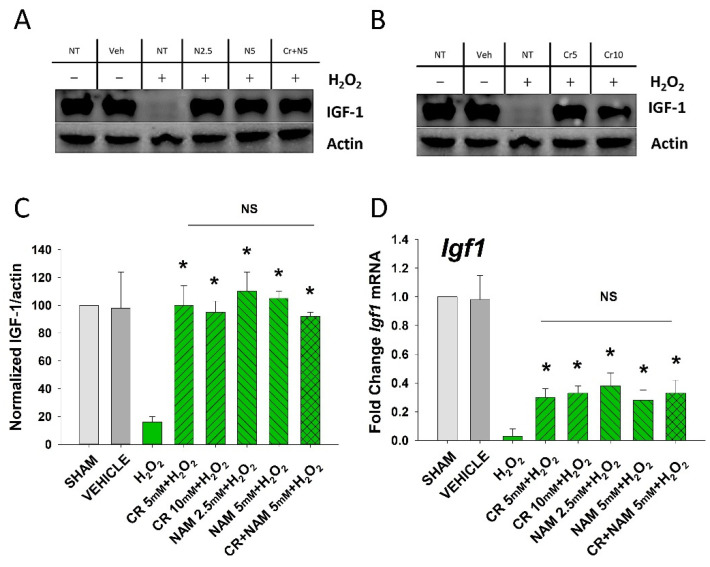
Effect of Cr and NAM on oxidant induced senescence-associated decreases in IGF-1 levels. DHF were exposed to various concentrations of Cr or NAM alone or combined (5 mM each), media alone (SHAM), or water vehicle (1%). After 24 h, the cells were exposed to 600 µM H_2_O_2_ for 2 h, followed by harvesting 72 h later (**A**,**B**). Examples of Western blotting indicating that H_2_O_2_ treatment decreased IGF-1 protein expression whereas pretreatment with (**A**) Cr and (**B**) NAM normalized IGF-1 protein levels; (**C**) graphical summary of (**A**,**B**) normalized (to actin) protein levels. Data sets of IGF-1 levels were quantified relative to actin and normalized to sham (**D**) IGF-1 mRNA was decreased by H_2_O_2_ but Cr and NAM pretreatment blunted the decrease of IGF-1 mRNA levels. Data sets of IGF-1 mRNA were quantified using the Livak method and normalized to sham. The data are mean ± SE from 3–4 separate experiments using duplicate samples. * Denotes statistically significant changes in the Cr/NAM treated levels in comparison to H_2_O_2_ alone. NS, the treatments compared are not statistically significantly different.

**Figure 5:**
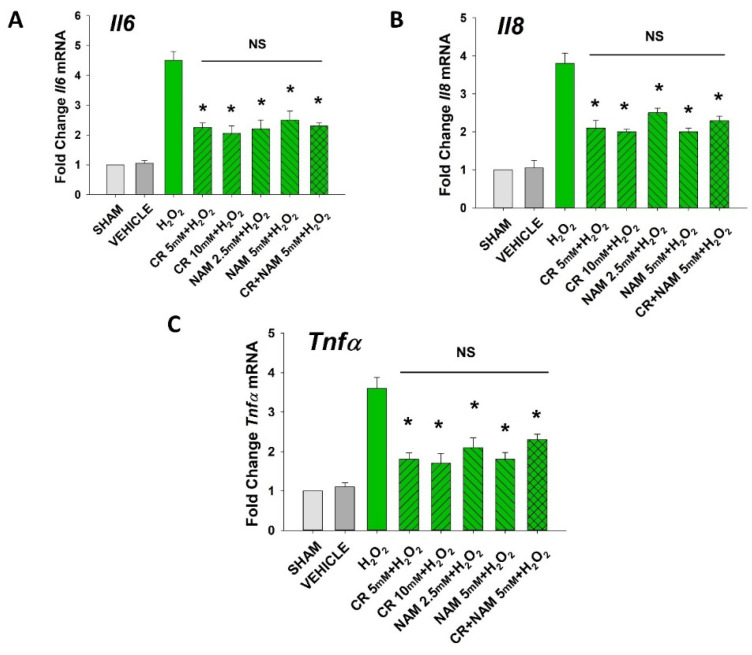
Effect of Cr and NAM on oxidant induced, senescence associated increased SASP cytokine mRNA levels. HDF were exposed to various concentrations of Cr or NAM alone or combined (5 mM each), media alone (SHAM), or water vehicle (1%). After 24 h, the cells were exposed to 600 µM H_2_O_2_ for 2 h, followed by harvesting 72 h later (**A**–**C**). The data are mean ± SE levels of mRNA from various cytokines normalized to actin from 3–4 separate experiments using duplicate samples. Data sets of SASP cytokines were quantified using the Livak method and normalized to sham. * Denotes statistically significant changes in the Cr/NAM treated levels in comparison to H_2_O_2_ alone. NS, the treatments compared are not statistically significantly different.

The senescent fibroblast secretes lower levels of IGF-1 and increased levels of SASP including IL-6, IL-8, and TNF-α [29,30]. The next studies were designed to define if Cr and NAM can normalize the abnormal senescence-associated cytokine profile generated in response to H_2_O_2_ treatment. As shown in Figure 4, oxidant exposure of DHF decreased IGF-1 mRNA and protein levels, which were somewhat normalized by Cr and NAM. Similarly, Cr and NAM pretreatment decreased the augmented mRNA expression levels of SASP cytokines IL-6, IL-8, and TNF-α associated with H_2_O_2_ treatment (Figure 5). 

### 3.4. Cr and NAM Do Not Protect When Given Post-Oxidant Stressor 

**Figure 6:**
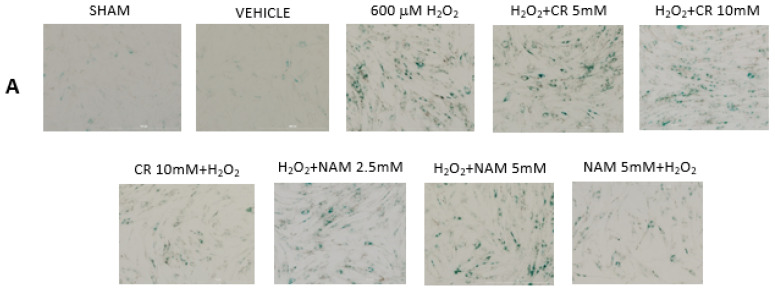

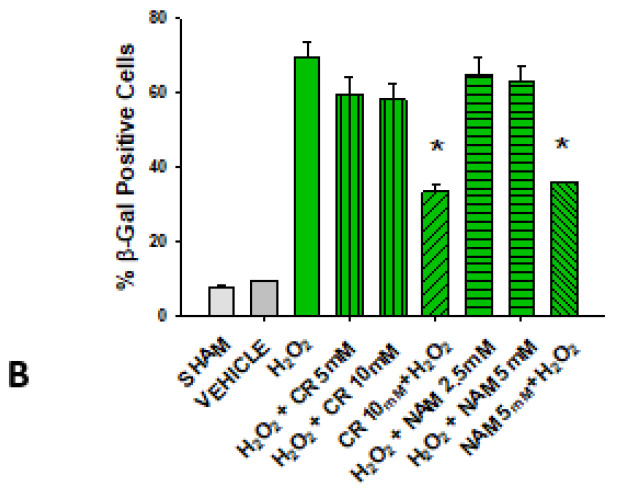
Effect of Cr and NAM post treatment on oxidant induced β-gal activity. DHF were treated with vehicle for 24 h, followed by exposure to 600 µM H_2_O_2_ for 2 h, followed by treatment with Cr or NAM. 72 h later cells were harvested for β-gal staining. In some experiments, DHF were pretreated with 10 mM Cr (Cr 5 mM + H_2_O_2_) or 5 mM NAM (NAM 5 mM + H_2_O_2_) before oxidant exposure as in Figure 2. (**A**) Representative pictures of β-gal staining in response to various treatments. (**B**) % β−gal positive cells in response to various treatments. The data are mean ± SE from 3–4 separate experiments using duplicate samples. * Denotes statistically significant changes in the Cr/NAM pretreated levels in comparison to H_2_O_2_ alone. None of the post-treatments were statistically significant in comparison to the H_2_O_2_ treatment alone.

The findings thus far indicate that fibroblasts pretreated with pro-energetics are protected against experimental senescence. The next studies were designed to test if these agents given post-H_2_O_2_ treatment can diminish the senescent response. To that end, DHF were treated with 600 µM H_2_O_2_ for 2 h followed by treatment with Cr or NAM or the combination for 72 h. At that time, β-gal staining was performed to assess numbers of senescent cells and mRNA of cytokines/IGF-1 measured. As denoted in Figure 6, neither Cr nor NAM posttreatment decreased the numbers of β-gal-positive cells generated in response to oxidant treatment. Similarly, Cr and NAM when given after.

**Figure 7:**
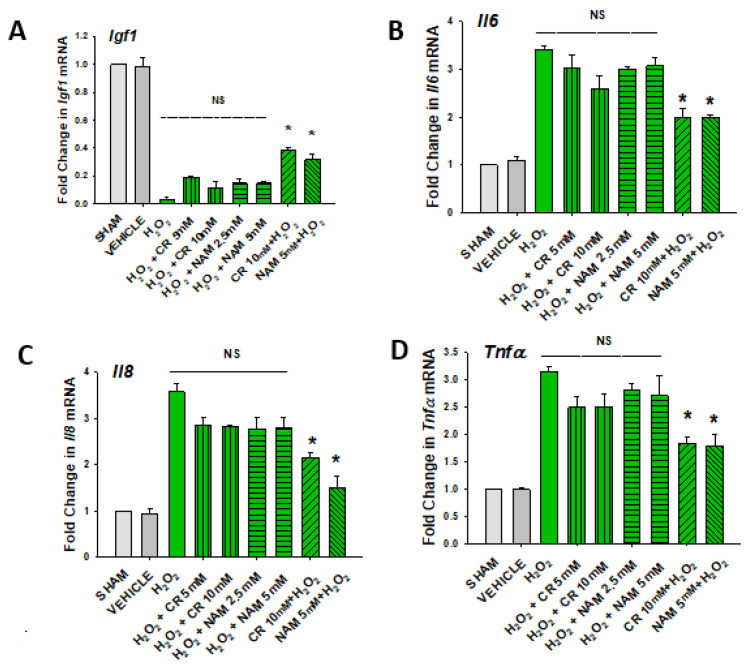
Effect of Cr and NAM posttreatment on oxidant induced IGF-1 and SASP mRNA expression. DHF were treated as in Figure 6, and IGF-1 or SASP cytokines measured (**A**–**D**). The data are mean ± SE from triplicate experiments . Data sets of SASP cytokines were quantified using the Livak method and normalized to sham. * Denotes statistically significant changes in the Cr/NAM treated levels in comparison to H_2_O_2_ alone. NS, the treatments compared are not statistically significantly different.

H_2_O_2_ did not modify augmented mRNA expression levels of SASP cytokines IL-6, IL-8, and TNF-α or increase IGF-1 mRNA levels (Figure 7). These studies indicate that the protective effects of pro-energetics necessitate that they are present before the pro-oxidative stressor.

### 3.5. Cr and NAM Block ROS Formation 

**Figure 8:**
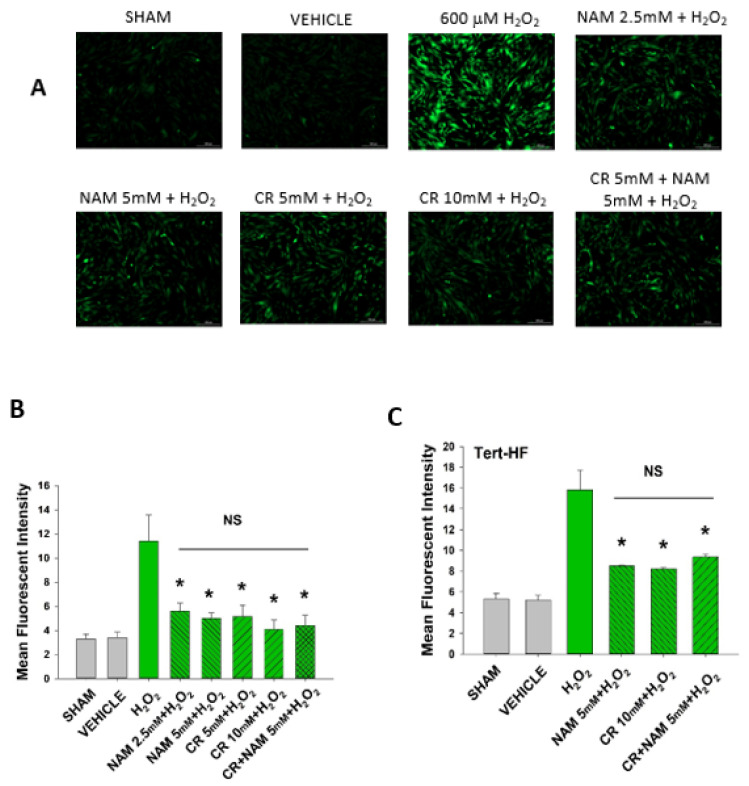
Effect of Cr and NAM pretreatment on oxidant induced ROS generation in DHF and Tert-HF. (**A**) Representative pictures of DCFH2-DA staining of vehicle versus 30 min 600 mM H_2_O_2_ treatment versus pretreatment for 24 h with CR or NAM alone or combined (5 mM each) in DHF. Cells were harvested at 30 min post oxidant treatment; (**B**,**C**) graphical representation of mean fluorescence intensity of (**B**) DHF or (**C**) Tert-HF. The data are mean ± SE from triplicate experiments. * Denotes statistically significant changes in the Cr/NAM treated levels in comparison to H_2_O_2_ alone. NS, the treatments compared are not statistically significantly different.

**Figure 9:**
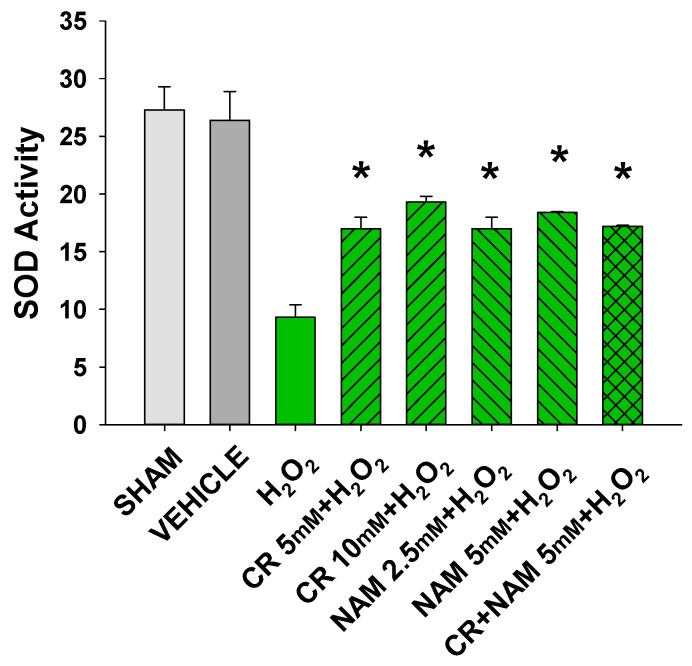
Effect of Cr and NAM pretreatment on SOD activity. DHF cells were exposed to various concentrations of Cr or NAM alone or combined, media alone (SHAM), or water vehicle (1%). Twenty-four hours later, the cells were exposed to 600 µM H_2_O_2_ for 30 min. SOD Assay kit-WST (Dojindo) was used measure SOD activity at that time. The data are mean ± SE from duplicate experiments using triplicate samples. * Denotes statistically significant changes in the Cr/NAM treated levels in comparison to H_2_O_2_ alone.

Increased cellular ROS is linked to induction of senescence [28,29]. The next studies were designed to test if Cr and NAM were exerting their protective effects via blocking the H_2_O_2_-induced increased ROS in DHF. As shown in Figure 8, H_2_O_2_ treatment increased ROS in DHF. Of importance, pretreatment with these pro-energetics attenuated the increased ROS response in these cells. Similar findings were noted in the TERT-immortalized human fibroblast cell line (Figure 8C). As expected, Cr and NAM pretreatment also blunted the H_2_O_2_-mediated decrease in SOD enzyme activity levels (Figure 9).

## 4. Discussion

Cellular senescence can be defined as the transformation of the cell into a state of arrested growth [29]. Senescence can be useful due to its tumor suppressive effects as cancer cells proliferate indefinitely. On the other hand, high levels of senescence may be deleterious to tissue regenerative capacity, growth, and differentiation [30,31]. In young organisms, where cellular metabolic pathways are well regulated, a short-term tumor suppressive exposure may be beneficial in combating cancers while not affecting the regenerative capacity of normal healthy cells. However, this may not be the case in older organisms. Cells in aged organisms may not function at their peak due to replicative exhaustion or stress induced DNA damage. These changes result in altered gene expression and hence affect the normal functioning of the cells. Such is seen in human dermal fibroblasts. Young (biochemically active) fibroblasts secrete connective tissues and other metabolic signals, one of them being IGF-1 [32]. This growth factor binds to its transmembrane receptors present on the keratinocytes, activating several pathways responsible for cell proliferation/metabolism/apoptosis. Data derived from using keratinocytes in culture as well as human clinical studies have provided significant premise for the novel paradigm that the increased susceptibility of aged skin to non-melanoma skin cancer involves fibroblast senescence [15]. In particular, the lack of dermal IGF-1 results in an inappropriate and pro-carcinogenic response of the overlying keratinocytes to UVB. 

There are at least four separate mechanisms by which activation of the keratinocyte IGF-1 receptor is protective. First, IGF-1 increases levels of DNA repair enzymes [32]. Second, keratinocytes not exposed to IGF-1 appear to carry out a more mutagenic form of DNA synthesis in response to UVB instead of the translesion synthesis DNA polymerase Pol eta [33]. Third, the protective pause in DNA synthesis following DNA damaging agents due to activation of a checkpoint signaling cascade is also diminished in IGF-1-depleted keratinocytes [34]. Thus, these three separate mechanisms result in the combination of less DNA repair capacity, repair with less fidelity, as well as replication of mutated DNA. Finally, IGF-1 receptor activation in keratinocytes will force the keratinocyte which cannot repair all of its DNA damage to undergo premature senescence, a protective mechanism. In contrast, keratinocytes deprived of IGF-1 are allowed to continue to proliferate which will result in continued mutations and eventual malignant degeneration [14,35,36].

Given this new paradigm of photocarcinogenesis involving dermal fibroblast senescence with resultant IGF-1 deficiency, therapeutics designed to prevent or treat fibroblast senesce could have use, especially in populations at high risk for skin cancer. Examples of agents currently studied for abilities to inhibit fibroblast senescence include extracts of microalga [37] or Akebia quinate fruit [38]. Additionally, Cr supplementation protects against mitochondrial mutagenesis as well as increases in matrix metalloproteinase-1 in response to UVA [39]. Data from other cell types provided some premise for testing the ability of pro-energetics Cr and NAM to block fibroblast senescence [17,24,25,38,40,41]. In particular, low levels of Cr in disease states including in kidney dialysis patients have been linked to overall poor health [41,42]. As pro-energetics such as Cr and NAM have popular uses from body building to anti-aging effects, and appear to have a relatively safe profile, these agents could be readily repurposed for NMSC prophylaxis.

Our study uses H_2_O_2_ as a means to induce stress and cause senescence. H_2_O_2_ is known to increase superoxide ions and free radicals, which increase ROS levels in the cell. Increased ROS levels can affect cell proliferation and induce DNA damage [40,41,43,44,45,46]. In our study, we performed an MTT assay to confirm the effect of H_2_O_2_ treatment on dermal fibroblast proliferation. Our findings suggest that a decline in cell proliferation was seen on Day 2 and Day 3 post H_2_O_2_ treatment, in 800 μM and 1 mM H_2_O_2_ exposures. Thus, our studies used 600 μM H_2_O_2_. No toxic effects of Cr and NAM at concentrations used in this study were noted. 

Both Cr and NAM pretreatment blocked the senescent response of H_2_O_2_ treatment. Senescence in the DHF was assayed using complementary biochemical changes of β-gal expression, p21 protein expression, as well as increased SASP cytokines and decreased IGF-1. However, exposure of these pro-energetics post-oxidant treatment did not result in appreciative protection from senescence. This finding as well as the fact that Cr and NAM prevented the increased cellular ROS in response to H_2_O_2_ treatment (Figure 8) indicate that these pro-energetics are exerting effects by blunting cellular ROS. It is worth mentioning that we did not note increased protection when combining Cr and NAM. This finding suggests that these agents likely use similar mechanisms. 

Though oxidant-induced senescence is a reasonable surrogate for the natural process of age-induced senescence, a more accurate model would potentially be to use senescence from multiple passages of the primary cells [47]. Future studies could test the ability of these pro-energetics to prevent replicative senescence. 

Regulated IGF-1 production is important to ensure normal cellular function, as well as to potentially prevent photo-carcinogenesis. In as much as the increased numbers of senescent fibroblasts found in aged skin result in a significant IGF-1 deficiency, strategies to prevent/and or treat this could have clinical use. In particular, our group recently reported that wounding of geriatric skin with fractionated laser resurfacing which upregulates active fibroblasts could prevent actinic neoplasia including NMSC [48]. Moreover, enzymatic treatment of photoaged skin with agents such as elastase could have beneficial effects [49]. Hence, potential uses of pro-energetics could be post-wounding to try to prevent further senescence of new fibroblasts generated in response to wounding or to retain physical properties in response to enzymatic treatments. An overall interpretation of the current studies is that topical pro-energetics could have clinical use to prevent, but not to treat fibroblast senescence. This could be a potential limitation on the use of these agents as therapies given the reality that most consumers would be more interested in an actual treatment for senescence rather than a preventative.

## 5. Conclusions

The present studies demonstrate that pretreatment of primary cultures of DHF with pro-energetics Cr and NAM protects against oxidant-induced senescence. These agents also appear to block the increased ROS associated with H_2_O_2_ treatment. These findings suggest that these agents could have use in preventing fibroblast senescence, which has been linked to the increased susceptibility of aged skin to nonmelanoma skin cancer. 

## Data Availability

Data is available upon request.
